# Long-term follow-up of retinal morphology and physiology after 2000 mg sildenafil overdose as a means of attempted suicide: a case report

**DOI:** 10.1186/s12886-022-02426-7

**Published:** 2022-05-12

**Authors:** Gen Miura, Takayuki Baba, Ryusuke Hashimoto, Shuichi Yamamoto

**Affiliations:** Department of Ophthalmology and Visual Science, Chiba University Graduate School of Medicine, Inohana 1-8-1, Chuo-ku, Chiba, 260-8677 Japan

**Keywords:** Sildenafil, Viagra, Overdose, Optical coherence tomography, Microperimetry, Electroretinogram

## Abstract

**Background:**

Few case reports have described sildenafil overdose, particularly ingestion of > 1000 mg, and overdose-induced changes in visual function remain unclear. We report retinal morphology, retinal sensitivity, and findings of electrophysiological evaluation over long-term follow-up in a case of sildenafil overdose (2000 mg).

**Case presentation:**

Our patient developed visual abnormalities in the paracentral visual field accompanied by photophobia, decreased contrast sensitivity, and difficulty distinguishing colors in both eyes, 8 hours after the sildenafil overdose. These symptoms did not improve throughout the course, and although abnormalities of retinal morphology and sensitivity, as well as the electroretinogram findings showed slight improvement, the patient did not recover completely at 6-month follow-up.

**Conclusions:**

We observed that high-dose sildenafil ingestion leads to retinal toxicity; the ocular abnormalities may persist for at least 6 months. Optical coherence tomography, Humphrey perimetry, microperimetry, and multifocal electroretinography are useful to quantitatively monitor temporal changes.

## Background

Sildenafil, a phosphodiesterase (PDE) 5 inhibitor, is widely used to treat erectile dysfunction and pulmonary arterial hypertension.

PDE-5 inhibitors inactivate PDE-6 and inhibit progression of the visual cascade, which results in blurred vision, blue-tinted vision, photophobia, abnormal electroretinogram (ERG) findings, high intraocular pressure (IOP), ischemic optic neuropathy, serous macular detachment, and retinal vascular occlusion. However, reportedly, these symptoms are typically transient [1]. A few studies have reported the effects of sildenafil on ocular tissues [2]; however, a limited number of studies have discussed sildenafil overdose (particularly doses > 1000 mg), and overdose-induced changes in visual function remain largely unknown. In this study, we report the retinal morphology, retinal sensitivity, and electrophysiological evaluation findings on long-term follow-up in a case of sildenafil overdose (2000 mg).

## Case presentation

### Initial evaluation 3 months after the overdose

A 23-year-old Asian man visited Chiba University Hospital with paracentral visual field impairment and blurred vision on exposure to bright light. He consumed 20 sildenafil (100 mg) tablets as a means of suicide, 3 months prior to presentation. He had been using 100 mg daily over 2 months before the overdose. After the overdose, he developed color blindness (particularly inability to distinguish white color), photophobia, and difficulty with the paracentral visual field. His color vision deficiency improved spontaneously; however, paracentral visual field impairment and blurred vision in a bright environment did not improve even 3 months after the overdose. He denied a personal or family history of ocular or autoimmune diseases. Laboratory investigations showed that serum alanine transaminase, aspartate transaminase, alkaline phosphatase, creatinine, blood urea nitrogen, and C-reactive protein levels were all within normal limits.

**Fig. 1:**
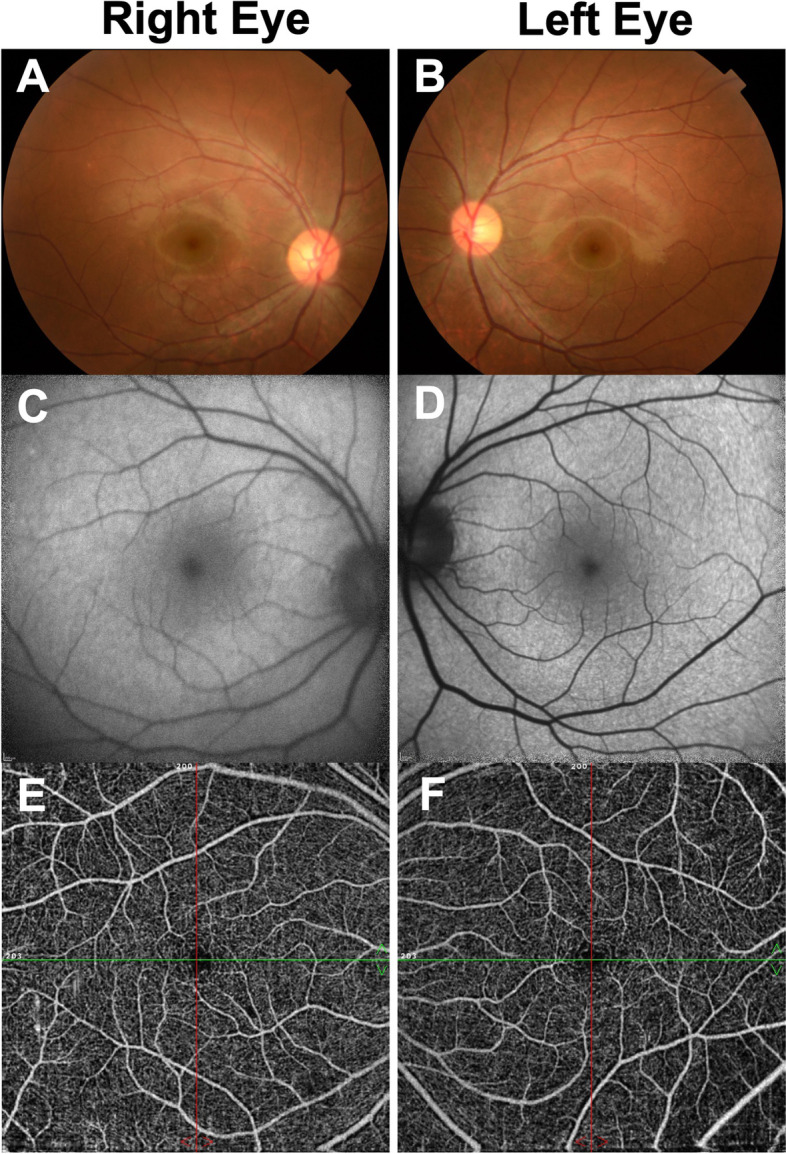
Fundus photographs (**A**, **B**) showing fundus autofluorescence (**C**, **D**), and optic coherence tomography angiography scan (**E**, **F**) showing findings at the initial visit 3 months after the overdose

**Fig. 2:**
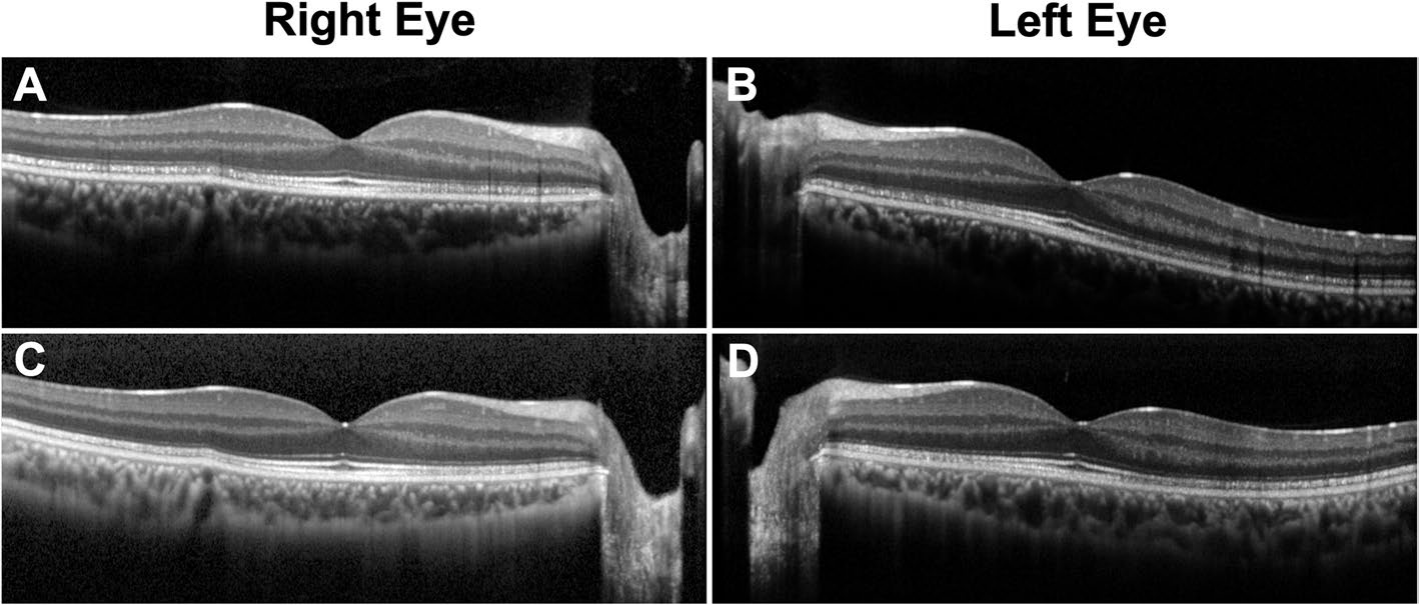
Optic coherence tomography images obtained 3 months (**A**, **B**) and 6 months (**C**, **D**) after the overdose

**Fig. 3:**
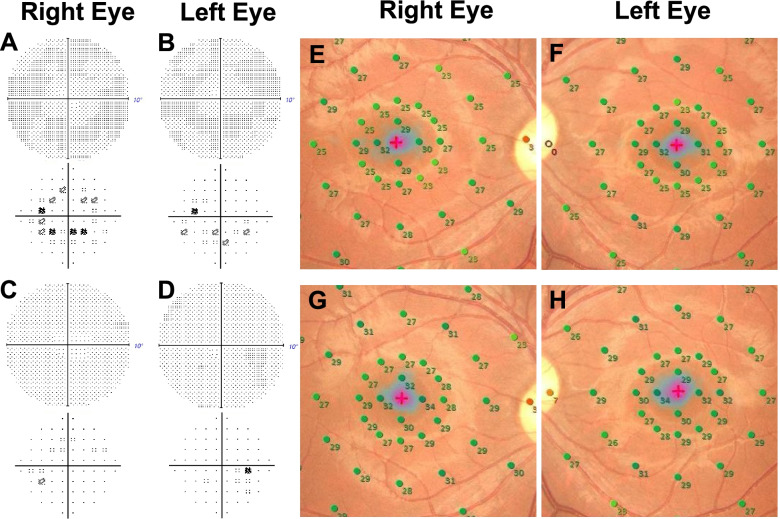
Grayscale and pattern deviation of the Humphrey field analyzer 10–2 program and results of MP3 microperimetry 3 months (**A**, **B**, **E** and **F**) and 6 months (**C**, **D**, **G** and **H**) after the overdose

**Fig. 4:**
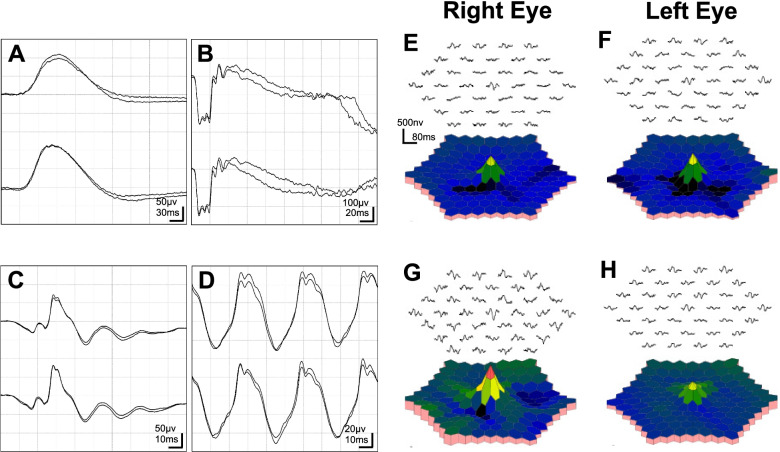
Full-field electroretinogram waveforms obtained 3 months after the overdose (**A**: dark-adapted 0.01, **B**: dark-adapted 3.0, **C**: light-adapted 3.0, **D**: light-adapted 30 Hz flicker). Multifocal electroretinogram waveforms at 3 (**E**, **F**) and 6 months (**G**, **H**) after overdose

Initial ophthalmological examination showed that his best-corrected visual acuity (BCVA) was 20/16 in both eyes, and IOPs were 10 and 13 mmHg in the right and left eyes, respectively. The pupillary light reflex was normal, and a relative afferent pupillary defect was not detected in either eye. The central critical flicker frequency was 43.3 Hz in the right and 44.1 Hz in the left eye. Slit-lamp examination of the anterior segment showed findings within normal limits. Bilateral fundoscopy did not reveal any clear abnormalities (Fig. 1A, B). Fundus autofluorescence (FAF) and optical coherence tomography (OCT) angiography did not reveal any apparent abnormalities (Fig. 1C, D, E, F). OCT showed mottling and discontinuity of the ellipsoid and interdigitation zones in areas other than the fovea (Fig. 2A, B). Choroidal thickness was within normal limits. The Humphrey Field Analyzer (HFA) 10–2 program showed a slight decrease in paracentral sensitivity in grayscale and pattern deviations, particularly in the right eye (Fig. 3A, B). MP-3 microperimetry revealed a decrease in bilateral paracentral sensitivity (Fig. 3E, F). We observed slight reduction in amplitudes of full-field photopic ERGs (Fig. 4C); however, the amplitudes of the scotopic, maximum combined, and flicker ERGs were within normal limits (Fig. 4A, B, D). Multifocal ERGs (mfERGs) were reduced, particularly in the bilateral paracentral areas (Fig. 4E, F).

### Findings 6 months after the overdose

The patient showed no improvement in subjective symptoms 6 months after the overdose. BCVA was 20/16 in both eyes, and IOPs were 11 and 13 mmHg in the right and left eyes, respectively. Discontinuity in the ellipsoid and interdigitation zones was slightly improved compared to that observed at 3 months; however, complete normalization was not observed (Fig. 2C, D). HFA 10–2 examination showed improved sensitivity to both grayscale and pattern deviations (Fig. 3C, D). MP-3 examination revealed improved retinal sensitivity, particularly at the paracentral 12 points (Fig. 3G and H). The mfERG amplitudes improved in nearly the entire area of the right eye and in the parafoveal area of the left eye (Fig. 4G, H).

## Discussion and conclusions

Usually, sildenafil is administered at a dosage of 25–100 mg/ day. Auso et al. reported sildenafil-induced visual adverse effects in 3–11% of men who received this drug at doses of 25–100 mg, in 50% of men who received 200 mg, and in 100% of men who received 600 or 800 mg of this drug [3–7]. These findings indicate that ocular adverse effects are dose-dependent. To our knowledge, this is the first case report that describes long-term follow-up (over 6 months) in a patient who overdosed on sildenafil (2000 mg).

A sildenafil overdose is associated with a variety of ocular side effects; compared with other ocular effects, reduction in the BCVA is less common. A 32-year-old woman with a history of primary pulmonary hypertension, who received oral sildenafil (60 mg/day) over 5 years showed a decrease in BCVA to 20/100 in the left eye and was diagnosed with parafoveal retinal pigment epithelium atrophy [8]. Another study reported a mild decrease in the BCVA to decimal visual acuity of 0.7 [9] and fractional visual acuity of 20/30 [10] at the initial visit in a patient who received high-dose sildenafil. In contrast, BCVA did not decrease in our patient, although the BCVA observed immediately after the overdose remains unknown in this case. Although studies have reported mydriasis after ingestion of 100 mg of sildenafil [11], the pupillary diameter and light reflex were normal in our patient at the initial 3-month examination. Serous foveal detachment has been reported after ingestion of 150–400 mg of sildenafil [12, 13]; however, our patient did not show retinal detachment.

Several case studies have reported thickening of the ellipsoid zone and increased choroidal thickness after high-dose sildenafil ingestion. Reportedly, these outer retinal abnormalities occur in the fovea in some patients but spare the fovea in others [9, 10, 14]. The central choroidal thickness and outer retinal blood flow were shown to increase significantly within 1 h of sildenafil ingestion [15]. In our case, OCT changes were observed in the parafoveal area without apparent thickening or thinning of the outer retina. The paracentral ellipsoid interdigitation zone discontinuity observed in our patient was similar to that previously reported in a patient who ingested a 2000 mg overdose [9], although the change was more widespread in our patient. The abnormality in choroidal thickness was unclear, and comparison between findings 3- and 6 months after the overdose did not show a clear difference in choroidal thickness, although findings immediately after the overdose were unknown. A previous study has reported OCT-documented retinal structural abnormalities that persisted for at least 1 year in a patient with oral sildenafil ingestion of 750 mg [14]. We could not exclude the possibility of persistent OCT-documented abnormalities for up to 1 year; therefore, we performed long-term follow-up in this case.

Some studies that have reported cases of sildenafil overdose have described a small range of hyperautofluorescence on FAF images [10, 16]. FAF did not show apparent abnormalities in either eye in our patient. We cannot rule out the possibility that abnormalities detected on FAF had disappeared at the time of initial examination 3 months after the overdose.

We observed improvement in HFA 10–2 results and in the sensitivity of mainly the paracentral 12 points on MP3 testing during the post-overdose period. The area of decreased sensitivity corresponded to the area of the retinal outer layer, which showed alterations, and sensitivity gradually improved at 6-month follow-up. The HFA10–2 and MP3 test findings were useful to detect abnormalities and to monitor changes over time. MP3 microperimetry may be more useful in cases of local injury because it can determine the correlation between the injured area and local sensitivity [17].

Previous studies have shown a transient rod and/or cone ERG amplitude reduction [18] and a slight peak time prolongation [19] at sildenafil doses < 300 mg. Only a few case reports have discussed electrophysiological assessment in cases of ingestion of higher doses. Yanoga et al. reported a slight decrease in the amplitude of the cone ERG, which is consistent with our result, although the amplitude of the foveal region on mfERGs was smaller than that observed in our study [16]. In our case, mfERGs were useful to confirm the time course of the abnormalities, as well as to determine the area of retinal dysfunction. Immunohistochemical analysis performed by Foresta et al. showed the PDE5 enzyme on bipolar and ganglion cells [20]; therefore, it is reasonable to conclude that sildenafil-induced visual impairment may be attributable not only to PDE6 inhibition at the photoreceptors but also to PDE5 inhibition in bipolar and ganglion cells. Electrophysiological evaluation can show the function of each retinal layer; further electrophysiological evaluation may be useful to confirm the mechanism underlying sildenafil-induced visual dysfunction.

Tear production is regulated by cyclic nucleotides; therefore, tear secretion abnormalities were initially considered an adverse effect of sildenafil. However, previous studies that investigated the effects of long-term sildenafil use on tear function did not report significant abnormalities [21]. No abnormal tear secretion was observed in our patient, which is consistent with the observations of a previous report.

Reportedly, subjective symptoms improved 21 days after ingestion of 100 mg [11] and 38 days after ingestion of 2000 mg of sildenafil [9]. In our patient, subjective symptoms persisted over 6 months after the overdose. However, the contributors to these disparities in findings remain unclear.

In summary, an overdose of sildenafil (2000 mg) was shown to cause abnormalities in retinal morphology and sensitivity, as well as ERGs, and mfERG alterations. These changes improved slightly at 6 months; however, complete normalization did not occur. Abnormalities of color vision, photophobia, and paracentral visual field defects did not improve completely at 6 months. Our findings showed that OCT, HFA10–2, microperimetry, and mfERGs were useful for quantitative monitoring of temporal changes. Further studies are warranted to determine the long-term outcomes after a sildenafil overdose to gain deeper insight into the toxicity and pathology of sildenafil.

## Data Availability

All the data supporting our findings are contained within the manuscript.

## Abbreviations

BCVA: Best-corrected visual acuity
ERG: Electroretinogram
FAF: Fundus autofluorescence
HFA: Humphrey field analyzer
IOP: Intraocular pressure
mfERG: Multifocal electroretinogram
OCT: Optical coherence tomography
PDE: Phosphodiesterase
